# Good outcome of tracheostomy in a COVID‐19 child with Joubert syndrome—Case report

**DOI:** 10.1002/ccr3.6973

**Published:** 2023-02-15

**Authors:** Veronica Epure, Doru Oprea, Dan Cristian Gheorghe

**Affiliations:** ^1^ “Carol Davila” University of Medicine and Pharmacy Bucharest Romania; ^2^ ENT Department “MS Curie” Hospital Bucharest Romania

**Keywords:** apnea, decannulation, Joubert syndrome, tracheostomy

## Abstract

Pediatric tracheostomy in COVID‐19 patients is a rarity. Joubert syndrome is a rare genetic disease, involving a lack of muscle control. We report the case of a child with Joubert syndrome and a severe form of COVID‐19 infection, in whom we performed tracheostomy in order to replace prolonged intubation and mechanical ventilation; successful decannulation was performed after 12 months. Successful decannulation is still possible in a child with severe comorbidities (Joubert syndrome) even if it might take much longer than in patients without comorbidities.

## Abstract 2

Pediatric tracheostomy in COVID‐19 patients is a rarity. Successful decannulation is still possible in a child with severe comorbidities (Joubert syndrome) even if it might take much longer than in patients without comorbidities.

## 1 INTRODUCTION

The global pandemic with coronavirus (COVID‐19) has led to a significant healthcare crisis. COVID‐19 infection is known to cause severe respiratory illness in adult population whereas children can be asymptomatic or mildly symptomatic carriers of this infection. In children with underlying comorbidity, this disease can be severe.

Use of tracheostomy in COVID‐19 patients can facilitate weaning from mechanical ventilation, but this is a high aerosole‐generating procedure with a high risk of disseminating the infection. There are few literature data on the subject, only a few authors reporting their experience with tracheostomy in adult COVID‐19 patients. Some authors recommend to perform tracheostomy not before 10 days after initiation of mechanical ventilation and intubation, in order to reduce the risk of spreading the infection to healthcare workers.^1^ The average time of intubation in COVID‐19 adults is 12–26 days^2^; the most common indication for tracheostomy in these patients is acute respiratory distress syndrome, followed by mechanical ventilation and failure to wean ventilation. The average time from tracheostomy to ventilator liberation is 5–20 days. In adults, the rate of decannulation after tracheostomy in COVID‐19 patients is low (3%).^3^

Joubert syndrome is a rare genetic disease (affecting 1 in 80–100,000 newborns), with autosomal recessive pattern of inheritance, generated by the agenesis of cerebellar vermis; it is also known as cerebello‐oculo‐renal syndrome and ″the molar tooth sign″ on cerebral MRI is a pathognomonic feature in this case.^4^, ^5^ The syndrome involves eye abnormalities, kidney disease, and skeletal abnormalities (extra fingers or toes, broad forehead, arched eyebrows, eyelid ptosis, hypertelorism, low set ears, triangle‐shaped mouth, cleft lip, and palate). Its most common features are lack of muscle control (ataxia, hypotonia, delay in motor development), abnormal breathing patterns (alternation of hyperpnea and apnea), and sleep apnea.^4^, ^5^, ^6^

We report the case of a child with Joubert syndrome and a severe form of COVID‐19 infection in whom open tracheostomy was performed, management and follow‐up. Written informed consent from the child's parents and approval from the Hospital's Ethics Committee were obtained.

## 2 CASE REPORT

Our patient, female, aged 14 months, presented to our hospital on November 2020 with fever and acute respiratory distress; COVID‐19 testing (PCR) returned positive. The patient was known with Joubert syndrome, being followed up by the Pediatrics Department in our hospital; the girl showed delayed motor development and hypotonia. The diagnosis of Joubert syndrome was based on genetic testing of the one‐month‐old infant (heterozygous deletion of the chromosomal region chr5:122700179–122,758,727, encompassing exons 2–19 of the CEP120 gene, and the carrier status of the CEP120 copy number variant is confirmed by NGS‐CNV analysis and confirmed by q‐PCR) and on brain MRI findings (corpus callosum and cerebellar vermis hypoplasia, enlarged IVth ventricle communicating largely with cisterna magna, molar tooth sign on axial sections).^4^, ^5^, ^6^ Polysomnography performed before the current admission showed periods of central apnea and irregular breathing patterns in this patient.

The evolution of the patient was severe, with acute hypercapnic respiratory distress and frequent episodes of central apnea. She was admitted to the ICU, intubated, and mechanically ventilated, on supportive treatment.

On day 13 (measured from the first day of positive PCR testing for COVID‐19), the medical team managing the case decided tracheostomy, in order to avoid laryngeal lesions due to prolonged intubation and to allow a better tracheobronchial management (December 2020).

**FIGURE 1:**
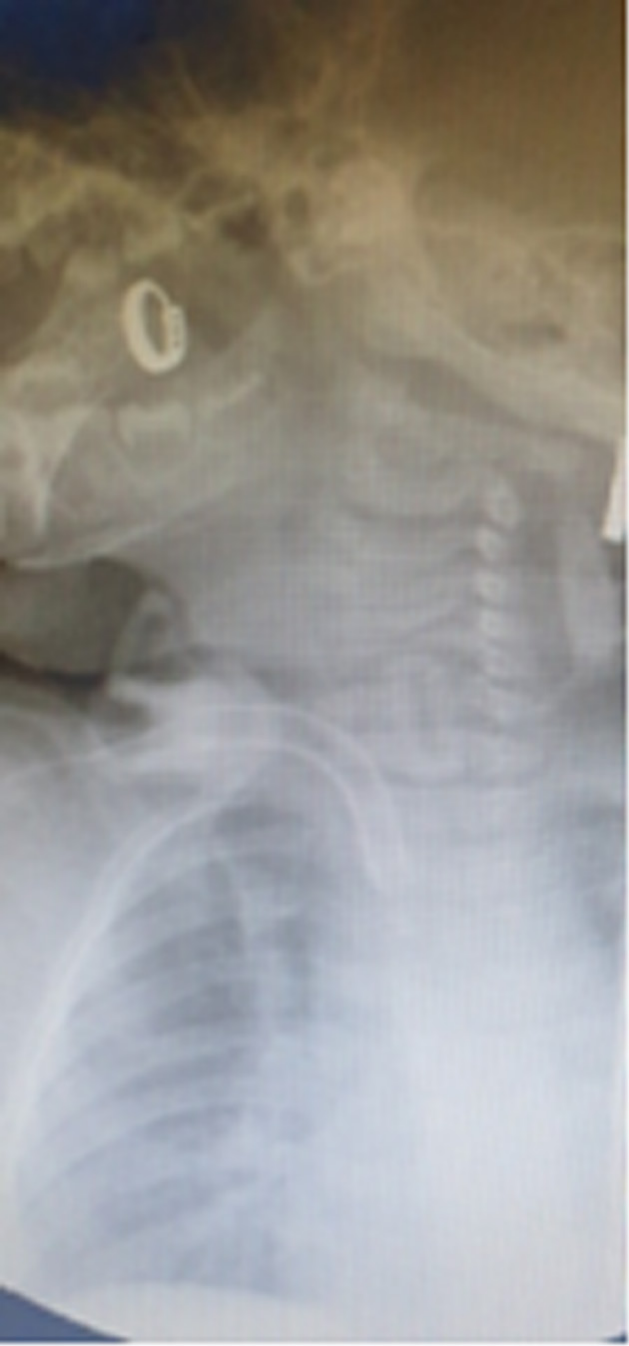
Verifying the position of the tracheostomy tube—tube in favourable position.

We performed opened T_3_‐T_4_ tracheostomy in the ENT Department OR; being a highly aerosole‐generating procedure, with increased risk of disseminating COVID‐19 infection, and all safety precautions were taken (protective PPE for the entire team, stop of assisted ventilation of the patient on opening the trachea until introducing the tracheostomy tube, using a cuffed tube, minimizing procedure time, avoiding electrocautery through fine dissection of tissues). Correct positioning of the tracheostomy tube was verified by X‐rays immediately after the procedure (tracheal fibreoscopy was avoided as another aerosole‐generating procedure) (Figure 1).

Evolution of the patient was favorable. She was soon weaned off ventilator, and she was discharged from hospital after 3 weeks on bilevel positive airway pressure.

**FIGURE 2:**
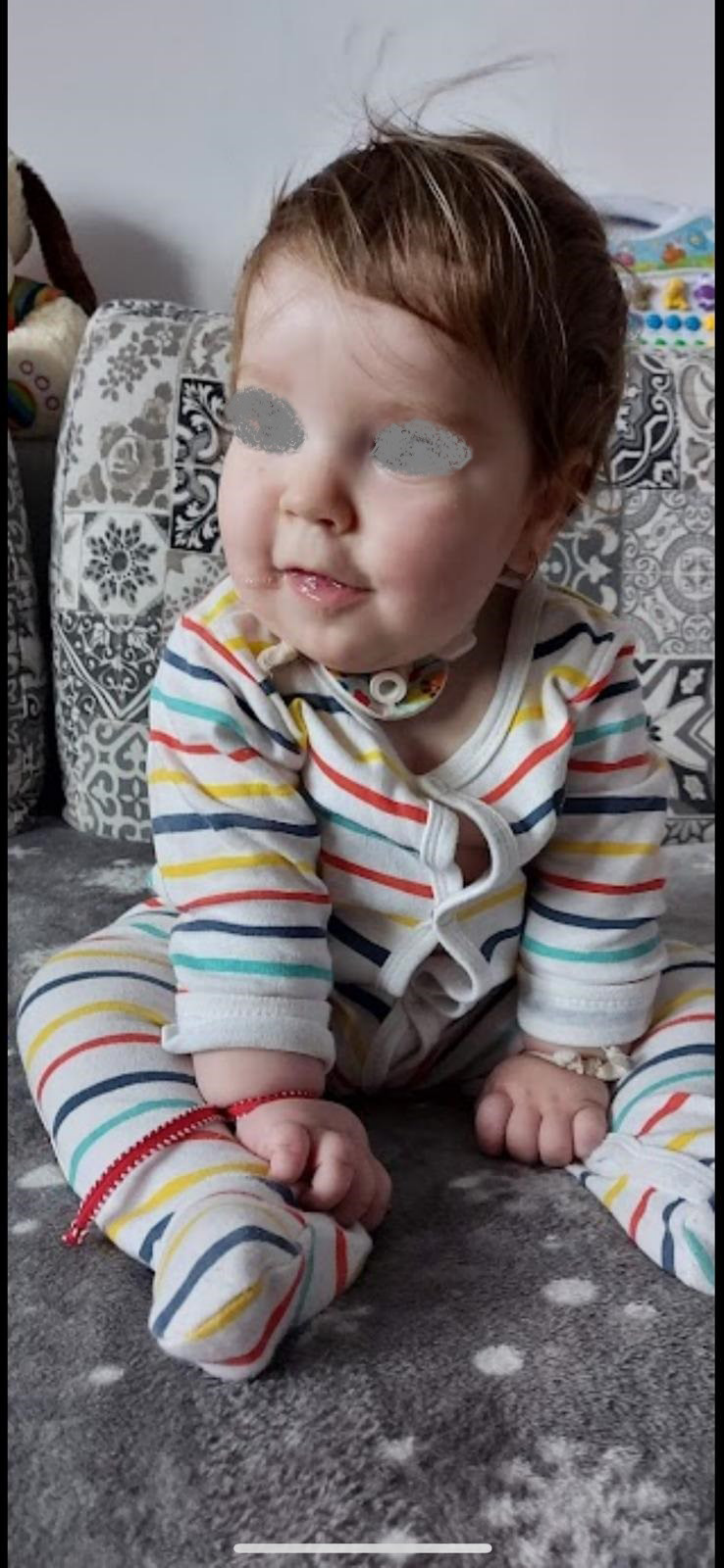
Child with Joubert syndrome at 6 months after tracheostomy—note the broad forehead, triangle‐shaped mouth, hypertelorism, and no other bone deformities.

The follow‐up of the patient included monitoring of the tracheostomy and changing the tracheostomy tube every 3 months, evaluation by the pediatrician, and polysomnography (Figure 2); she was discharged while on ventilator, but by 5 months after tracheostomy the patient was breathing spontaneously with no need of assisted ventilation; the deglutition was almost normal (with few episodes of aspiration while drinking clear fluids persisting for 2 months).

After 12 months after tracheostomy, we decided decannulation of the patient, having a stable healthy patient with no more deglutition problems and thus no risk of aspiration. Following the protocols in our department, we performed awake fibre laryngoscopy and then laryngo‐tracheoscopy under general anesthesia; the larynx and trachea were normal. After awakening the patient, we performed one‐step decannulation of the patient. She stayed for 24 h under supervision on our ward (polysomnography was performed during the first night following decannulation, showing regular breathing pattern, no central apnea); then, the patient was discharged home with good evolution during the next 1‐year follow‐up.

## 3 DISCUSSION

Joubert syndrome is a rare genetic affliction, involving a delay in motor development of the child and abnormal breathing patterns (alternation of hyperpnea and apnea).^4^, ^5^, ^6^ Additionally affected by COVID‐19, our patient with Joubert syndrome rapidly evolved to acute hypercapnic respiratory distress and frequent episodes of central apnea, needing urgent intubation and mechanical ventilation.

Performing tracheostomy in intubated COVID‐19 adult patients was quite common procedure during the pandemic but was exceptionally used in children^4^, ^8^; children with severe forms of COVID‐19 infection were rare.

Outcome after tracheostomy in COVID‐19 patients is usually unfavourable; the rate of decannulation after tracheostomy in COVID patients is low (13%), intubated adult patients have a poor prognosis, with few of them surviving extubation.^3^, ^7^ Tracheostomies provide several advantages: less requirement of sedation compared with oral intubation, less medical support, and decreased dead space of the airway.

Tracheostomy reports in children with COVID‐19 infections are scarce,^8^, ^9^, ^10^ as severe forms of COVID‐19 are rarer in children compared with adults.

Children are less susceptible to COVID‐19 infections and typically have milder manifestations than adults. In the United States, 2% of pediatric COVID‐19 cases require hospitalization, and up to 0.03% result in death (lower mortality rate compared with adults). Approximately 0.5%–18% of hospitalized children with COVID‐19 need ventilatory support and 2.1% need intubation. In a study on 48 children with COVID‐19 from ICU, 38% of them needed intubation and mechanical ventilation, 89% of these had severe comorbidities; out of intubated children, approximately 30% needed tracheostomies. Mortality rate among intubated children with COVID‐19 is 4.2% according to a multicenter study.^11^

In addition to less severe infections, several children‐specific considerations contribute to this findings. First, the pliable cartilaginous airway, especially among neonates, tolerates longer intubation time and does not justify earlier tracheostomy to minimize airway stenosis. Second, pediatric open tracheostomy is a high‐risk procedure with placement not amenable to percutaneous techniques and requiring experience. Finally, hospitalization after tracheostomy in children is often prolonged due to caregiver training and discharge barriers. Taken together, these factors explain why teams caring for children might cautiously approach decisions to proceed with tracheostomy.^8^, ^9^, ^10^ Complications of tracheostomy procedure in COVID‐19 patients are as follows: increased intraoperatory hemorrhage due to anticoagulant treatment (16.6%–24%), ulcers and granulations around stoma and in the larynx, tracheomalacia, crust formation inside the tracheal tube with concomitant blockage, air leak, and accidental decannulation.^8^, ^10^

Due to reduced number of cases, decannulation rate in children remains unestimated; one study^8^ on 18 children with tracheostomies performed during severe COVID‐19 infection reports 23.5 days (16–31 days) as the mean time between tracheostomy and decannulation and approximately 50% decannulation rate in children.

Duration of intubation (before tracheostomy) in children is 7–26 days (mean value 10 days)^8^, ^9^; tracheostomy is not associated with significantly better outcome in severe COVID‐19 children; the average time from tracheostomy to ventilator liberation was 11.8 ± 6,9 days^9^; decannulation was successful in approximately 35%‐50% of patients. Tracheostomy duration (until decannulation) is usually 17–41 days according to different authors; tracheostomized patients have a high risk of nosocomial infections and prolonged ICU and hospital stay.

The patient in our study had several comorbidities contributing to respiratory distress. A meta‐analysis of 285,014 children with COVID‐19 found severe disease developing in 5.1% with comorbidities compared with 0.2% without.^11^, ^12^ Joubert Syndrome involves a lack of muscle control and abnormal breathing patterns (alternation of hyperpnea and apnea), and symptoms were enhanced by acute COVID‐19 induced respiratory distress, leading the way to intubation and mechanical ventilation of the child. The decision to perform a tracheostomy in this child was taken in order to avoid laryngotracheal lesions due to prolonged intubation and to offer good airway access and a good option in order to avoid food aspiration risk after hospital discharge in a patient with neuromuscular problems. Decannulation took extremely long in our patient (12 months, much longer than the usual 1–3 months^8^, ^9^ because of her comorbidities; although the child was released from hospital after 3 weeks while on ventilator (on bilevel positive pressure), by 5 months after tracheostomy the child needed no more assisted ventilation but deglutition problems (episodes of aspiration of liquids) still persisted for 2–3 months due to neuromuscular deficit of the child with Joubert syndrome); a few intercurrent mild respiratory infections occurring in the few following months, together with dysphagia, were reasons of postponing decannulation until the patient was in a good and stable state.

Tracheostomy proved to be a safe procedure in our pediatric patient, with no spread of COVID‐19 infection to the care team, no postdischarge management problems, and no laryngotracheal sequels 2 years after its performance.

## AUTHOR CONTRIBUTIONS

**Veronica Epure:** Conceptualization; data curation; formal analysis; methodology; writing – original draft; writing – review and editing. **Doru Oprea:** Data curation; formal analysis; validation; writing – original draft; writing – review and editing. **Dan Cristian Gheorghe:** Formal analysis; methodology; project administration; resources; software; supervision; writing – original draft; writing – review and editing.

## FUNDING INFORMATION

None.

## CONFLICT OF INTEREST STATEMENT

None.

## INFORMED CONSENT

Written informed consent from the child's parents and approval from the Hospital's Ethics Committee were obtained to publish this report in accordance with the journal's patient consent policy.

## Data Availability

Data available on request due to privacy/ethical restrictions.
